# Measuring Activity of Native Plant Sirtuins - The Wheat Mitochondrial Model

**DOI:** 10.3389/fpls.2018.00961

**Published:** 2018-07-05

**Authors:** Mario Soccio, Maura N. Laus, Michela Alfarano, Donato Pastore

**Affiliations:** Dipartimento di Scienze Agrarie, degli Alimenti e dell’Ambiente, Università di Foggia, Foggia, Italy

**Keywords:** plant sirtuin, wheat mitochondria, bioluminescent sirtuin activity assay, HTRF^®^ sirtuin activity assay, resveratrol, quercetin

## Abstract

Sirtuins are NAD^+^-dependent deacetylase enzymes that have gained considerable interest in mammals for their recognized importance in gene silencing and expression and in cell metabolism. Conversely, knowledge about plant sirtuins remains limited, although a sirtuin-mediated regulation of mitochondrial energy metabolism has been recently reported in *Arabidopsis*. However, so far, no information is available about direct measurement of intracellular plant sirtuin activity, i.e., in cell extracts and/or subcellular organelles. In this study, a novel approach was proposed for reliable evaluation of native sirtuin activity in plant samples, based on (i) an adequate combinatory application of enzymatic assays very different for chemical basis and rationale and (ii) a comparative measurement of activity of a recombinant sirtuin isoform. In particular, two sirtuin assays were applied, based on bioluminescence emission and Homogeneous Time-Resolved Fluorescence (HTRF^®^) technology, and the human SIRT1 isoform (hSIRT1) was used for comparison. For the first time in plants, this new approach allowed measuring directly a high and nicotinamide-sensitive sirtuin activity in highly purified mitochondrial fraction obtained from durum wheat (WM). WM-sirtuin activity was 268 ± 10 mU⋅mg^-1^ protein, as measured by HTRF^®^ assay, and 166 ± 12 ng hSIRT1 eq.⋅mg^-1^ protein, as evaluated by the bioluminescent assay and calculated on the basis of the hSIRT1 calibration curve. Moreover, effects of resveratrol and quercetin, reported as potent hSIRT1 activators, but whose activation mechanism is still debated, were also studied. No effect of resveratrol was found on both WM-sirtuin and hSIRT1 activities, while only a slight increase, up to about 20%, of hSIRT1 activity by quercetin was observed. In the whole, results of this study indicate that WM may represent a good system for studying native plant sirtuins. In fact, the high yield of purified WM and their high sirtuin activity, together with use of microplate readers, allow performing a large number of measurements from the same preparation, so qualifying the approach for application to large-scale high-throughput screening. Moreover, WM may also represent an excellent tool to investigate physiological role and modulation of plant sirtuins under experimental conditions more physiologically relevant with respect to recombinant purified enzymes.

## Introduction

Sirtuins are ubiquitous enzymes belonging to class III of histone deacetylases that catalyze the specific NAD^+^-dependent deacetylation of ε-*N*-acetyl lysine residues of both histones and non-histone proteins to produce NAM, 2′-*O*-acetyl-ADP-ribose and the deacetylated polypeptide (Michan and Sinclair, 2007; Chung et al., 2010; Haigis and Sinclair, 2010; Szućko, 2016). Additionally, some sirtuins may also exhibit NAD^+^-dependent desuccinylation, demalonylation and defatty-acylation of lysine residues, as well as mono-ADP-ribosyltransferase activity (Szućko, 2016 and references therein). Sirtuin-mediated protein deacetylation represents a highly regulated post-translational modification having a strong impact on protein functions, enzyme activities, as well as on protein–protein and protein–DNA interactions (König et al., 2014). Multiple substrates of sirtuin activity have been identified including numerous regulatory proteins involved in many metabolic processes and defense mechanisms in response to stress (Chung et al., 2010).

In mammals, seven sirtuin isoforms (SIRT1-7) have been identified showing nuclear (SIRT1/6/7) (Michan and Sinclair, 2007; Haigis and Sinclair, 2010; Bai and Zhang, 2016), mitochondrial (SIRT3/4/5) (Gertz and Steegborn, 2010; Bell and Guarente, 2011) and cytosolic (SIRT2) (North et al., 2003; Black et al., 2008) localization. As for biological implications, animal sirtuins have been shown to regulate a wide variety of processes. In particular, for the best-characterized human SIRT1 (hSIRT1) an important role in regulating pathogenesis of diabetes, obesity, cancer, as well as neurodegenerative, cardiovascular, chronic renal and pulmonary diseases is reported (Chung et al., 2010).

Concerning plant sirtuins, only few studies can be retrieved from literature and regard only few species. Compared to other eukaryotes, plant have relatively fewer sirtuin-related genes coding for nuclear (SRT1) (Huang et al., 2007; Cucurachi et al., 2012; Zhao et al., 2015) and mitochondrial (SRT2) (Chung et al., 2009; Cucurachi et al., 2012; König et al., 2014) isoforms, although a nuclear/cytosolic localization has been obtained for SRT2 in tomato (Zhao et al., 2015). The knowledge about sirtuin functions in plants is still limited. Plant sirtuins were suggested to have a protection role against genome instability and cell oxidative damage required for plant cell growth (Huang et al., 2007), as well as to be implicated in gametogenesis, development and ripening of fruits (Zhao et al., 2015), leaf senescence and regulation of photosynthetic activity (Cucurachi et al., 2012) and auxin signaling (Grozinger et al., 2001; Hollender and Liu, 2008). Interestingly, a relevant role in fine-tuning of mitochondrial energy metabolism has been recently demonstrated for *Arabidopsis* SRT2 (König et al., 2014). Recently, in *Arabidopsis* mitochondria the existence of the first histone deacetylase of class I (HDA14) was also reported (Hartl et al., 2017).

Given their relevant role in a wide variety of cellular processes, sirtuins have been extensively investigated in last decades. In particular, mammalian sirtuins have been received increasing attention as important attractive drug targets and some modulators of sirtuin activity have been shown to have very promising therapeutic value for treating many human chronic and degenerative diseases (Chung et al., 2010; Sánchez-Fidalgo et al., 2012; Hubbard and Sinclair, 2014).

Much of current knowledge about functional characterization and modulation of sirtuin-dependent deacetylase activity in biological systems is inferred from gene-expression studies that are often not accompanied by enzymatic activity measurements (Chung et al., 2010). It should also be considered that information available about sirtuin activity and modulation is generally referred to purified recombinant enzymes. Although catalytic activity and effects of modulators highlighted on purified enzyme should be confirmed in cellular systems, to date only few data can be retrieved from literature reporting direct assessment of sirtuin activity in yeast/animal cell extracts (de Boer et al., 2006; Chai et al., 2017; Rogacka et al., 2018). In plants, so far, sirtuin-dependent deacetylase activity has never been directly measured in cell lysates. Only one paper concerning plant sirtuin activity is available, in which activity was assessed using *Arabidopsis* recombinant SRT2 proteins overexpressed and purified from *Escherichia coli*, as well as an indirect measurement was obtained by assessing changes in lysine acetylation levels of mitochondrial proteins in knockout lines compared to the wild type (König et al., 2014).

Moreover, to the best of our knowledge, no information is available about sirtuin activity assessment in subcellular organelles, both in animal and plant systems. The difficulty of carrying out direct sirtuin activity measurements in cell/cell lysates can depend on the complexity of the commonly employed enzymatic tools. Most of these assays imply indirect measurements that can be influenced and distorted by potentially interfering physiological compounds in the biological extract. The use of these indirect assays can further affect results leading to a compromised data interpretation in the study of direct modulation of sirtuin activity by chemical compounds. For example, the use of a fluorimetric assay exploiting peptide substrates conjugated to the AMC fluorophore has led to controversies on the mechanism of SIRT1 activation by resveratrol and its analogs (Borra et al., 2005; Pacholec et al., 2010).

In the light of above reported observations, appropriate assays for measuring sirtuin enzymatic activity should be carefully selected. Firstly, these assays should be able to achieve reliable and reproducible results and to be easily applicable directly on the “native” enzyme, i.e., on the enzyme in its biological environment (within the cell/subcellular organelle). Moreover, assays should be based on methodological approaches that are (i) able to provide an appropriate quantification of sirtuin activity to allow comparison among different experimental conditions and systems, and (ii) as free as possible from false positives or negatives due to interfering molecules in the reaction mixture/biological sample. With respect to the last point, an appropriate combinatory application of different methods can be an effective tool for highlighting undesirable interfering effects.

In the light of this, in the present study, sirtuin activity was measured by using two enzymatic assays: a bioluminescent assay (SIRT-Glo^TM^, Promega) and a Homogeneous Time Resolved Fluorescence (HTRF^®^)-based assay (HTRF^®^ SIRT1, Cisbio). They were chosen as very different in terms of reaction mechanisms, experimental conditions, methodologies used for monitoring the reaction progress and for quantifying results, so that their comparison could clearly unmask possible interfering effects. For both methods, parallel assessment of activity of a commercially available recombinant sirtuin isoform was carried out. In particular, a recombinant hSIRT1 was used, since it is the best-characterized sirtuin in terms of deacetylase activity and modulation by phenolic compounds, as well as the well-studied human sirtuin for its relevant role in regulating critical metabolic and physiological processes (Chung et al., 2010; Hubbard and Sinclair, 2014). This experimental approach was applied to measure for the first time a “native” plant sirtuin activity. In particular, mitochondrial sirtuin was studied, since this is the best-characterized plant sirtuin showing significant relevance in regulating mitochondrial energy metabolism (König et al., 2014). To this aim, durum WM were used, since an isolation procedure providing high yield of pure and intact organelles is available (Pastore et al., 1999) and bioenergetics aspects of these mitochondria have been investigated in detail (Pastore et al., 2007; Laus et al., 2008, 2011; Soccio et al., 2010, 2013, Trono et al., 2011, 2013).

Moreover, by using the developed approach, the effect on both hSIRT1 and WM-sirtuin activity of two polyphenols, such as resveratrol and quercetin, was evaluated. This in order (i) to assess in our experimental conditions the effect of these compounds, reported as potent natural SIRT1 activators (Howitz et al., 2003), but whose activation mechanism has been the subject of an intense debate (Hubbard and Sinclair, 2014), and (ii) to evaluate a possible role of these phytochemicals also as physiological modulators of plant sirtuin activity.

## Materials and Methods

### Chemicals and Plant Materials

All chemicals at the highest commercially available purity were purchased from Sigma-Aldrich Co. (St. Louis, MO, United States). The SIRT-Glo^TM^ and the HTRF^®^ SIRT1 assays were purchased from Promega Co. (Madison, WI, United States) and Cisbio (Bedford, MA, United States), respectively.

Oligomycin was dissolved in ethanol; resveratrol and quercetin were dissolved in ethanol or dimethylsulfoxide (DMSO) for sirtuin activity measurements using the SIRT-Glo^TM^ or the HTRF^®^ SIRT1 assays, respectively.

Certified seeds of durum wheat (*Triticum durum* Desf., cv Ofanto) were kindly supplied from the CREA-Cereal Research Centre (Foggia, Italy).

### Wheat Mitochondria (WM) Isolation

Wheat mitochondria were purified from 72-h-old etiolated seedlings, as reported in Pastore et al. (1999) with minor modifications. The grinding and washing buffers were: (i) 0.5 M sucrose, 4 mM cysteine, 1 mM EDTA, 30 mM Tris-HCl (pH 7.50), 0.1% (w/v) defatted BSA, 0.6% (w/v) PVP-360; and (ii) 0.5 M sucrose, 1 mM EDTA, 10 mM Tris-HCl (pH 7.40), 0.1% (w/v) defatted BSA, respectively. Washed mitochondria were subjected to an isopycnic centrifugation in a self-generating density gradient containing 0.5 M sucrose, 10 mM Tris-HCl (pH 7.20) and 28% (v/v) Percoll (colloidal PVP coated silica, Sigma-Aldrich) in combination with a linear gradient of 0% (top) to 10% (bottom) PVP-40 (Moore and Proudlove, 1987) to obtain the purified mitochondrial fraction. For determination of NAM-sensitive sirtuin and marker enzyme activities in the different fractions obtained in the course of WM purification (**Table 3**), purified mitochondria were recovered, as well as the initial homogenate and the combined pellets obtained by the first and third centrifugations according to protocol described in Trono et al. (2013).

Protein content was determined by the method of Lowry modified according to Harris (1987) using BSA as a standard.

WM showed high intactness of inner and outer membranes and a good functionality, as evaluated by means of fluorimetric measurements of electrical membrane potential according to Pastore et al. (1999), Laus et al. (2008, 2011), Soccio et al. (2013).

### Enzymatic Assays

#### Sirtuin Assays

##### SIRT-Glo^TM^ assay (Promega)

**FIGURE 1:**
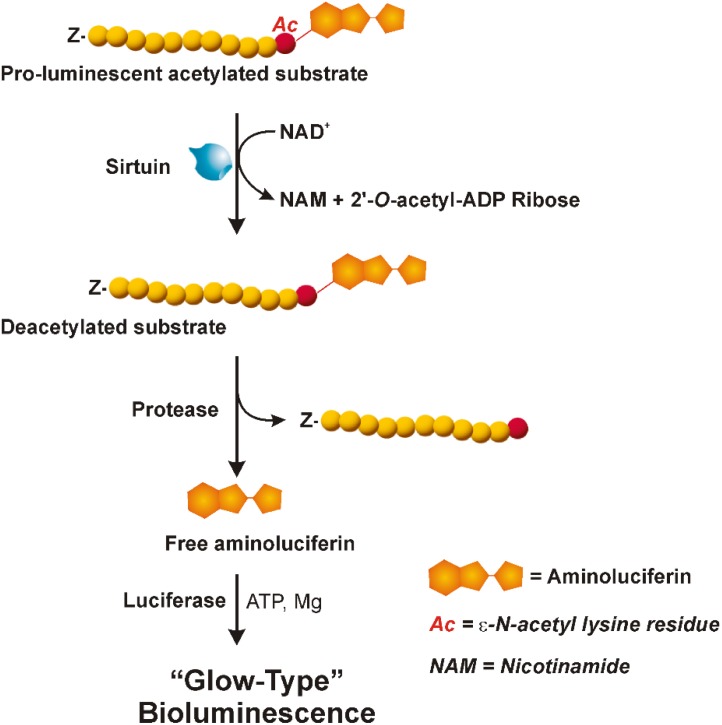
Bioluminescent assay (SIRT-Glo^TM^, Promega). The three reactions involved in the sirtuin assay are shown: NAD^+^-dependent deacetylation by sirtuin of the pro-luminescent substrate; cleavage by a specific protease of the deacetylated substrate resulting in aminoluciferin release; luciferase reaction producing a stable and persistent emission of light. “Z” represents an amino-terminal blocking group that protects the substrate from non-specific cleavage. All three enzymatic events occur in coupled. For more details see the text. Adapted from Technical Manuals of SIRT-Glo^TM^ Assay and Screening System (Promega) and HTRF^®^ SIRT1 assay (Cisbio).

As shown in **Figure 1**, the assay uses a pro-luminescent substrate containing an acetylated lysine attached to aminoluciferin. In particular, it is a sirtuin-optimized amino acid sequence based on a consensus sequence derived from p53; it also contains an amino-terminal blocking group (Z) that prevents non-specific cleavage. The NAD^+^-mediated deacetylation by sirtuin produces NAM, 2′-*O*-acetyl-ADP ribose and the deacetylated peptide. This last is then cleaved by a specific protease resulting in aminoluciferin release. The free aminoluciferin is then quantified using an ATP-dependent firefly luciferase reaction to produce a stable and persistent “glow-type” light emission that is proportional to sirtuin deacetylase activity.

Measurements were performed using a CLARIOstar microplate reader (BMG Labtech, Ortenberg, Germany) and 96-well plates and the manufacturer’s protocol was applied with slight modifications. The reaction mixture (final volume of each well: 200 μL) consisted of the SIRT- Glo^TM^ Buffer and the sample, i.e., the sirtuin enzyme source (hSIRT1, WM, homogenate and combined pellets). For sirtuin activity measurements in WM, the reaction mixture also contained: 0.1% Triton X-100, in order to lyse mitochondria so releasing mitochondrial sirtuin; 30 μM P^1^,P^5^-di(adenosine-5′) pentaphosphate (Ap5A) and 4 μg oligomycin, able to inhibit adenylate kinase and ATPase, respectively, in order to avoid consumption by mitochondrial metabolism of ATP, necessary for the firefly luciferase reaction. It was preliminary checked that Triton X-100, Ap5A and oligomycin had no significant effect on sirtuin activity detection system. The reaction was started by adding the SIRT-Glo^TM^ Reagent (prepared according to the manufacturer’s protocol by combining the SIRT-Glo^TM^ Substrate Solution and the Developer Reagent and containing substrate, NAD^+^, ATP, protease and luciferase) and monitored by recording the bioluminescence increase at 25°C, until to steady-state signal. The maximum signal expressed as RLU was determined and “signal to noise ratio” was calculated, i.e., the ratio between luminescent signal of sample and that of no-sirtuin (buffer background) control. The reaction rate was also calculated as the highest slope of the experimental curve and expressed as RLU⋅min^-1^.

For both hSIRT1 and WM sirtuin activity measurements, three different experiments were carried out. In each experiment determinations were carried out in triplicate by analyzing at least four different amounts of sample. A linear dependence of both signal to noise ratio and RLU⋅min^-1^ on the protein amount of hSIRT1 or WM was verified by linear regression analysis of data. WM-sirtuin activity was obtained by comparing the slope derived by linear regression analysis with that of the curve relative to hSIRT1 and expressed as hSIRT1 equivalent (ng hSIRT1 eq. mg^-1^ of WM protein). In experiments aimed at evaluating the effect of resveratrol and quercetin on sirtuin activity, the compound to be tested was added to the reaction mixture and incubated for 30 min before starting the reaction. In order to detect false positives due to inhibition of protease and/or luciferase, measurements were also carried out using a non-acetylated control substrate under the same experimental conditions as the acetylated SIRT-Glo^TM^ Substrate. Since oligomycin, resveratrol and quercetin were dissolved in ethanol that can affect sirtuin activity, a constant volume of ethanol (5 μL) was maintained in the reaction mixture.

##### HTRF^®^ SIRT1 assay (Cisbio)

**FIGURE 2:**
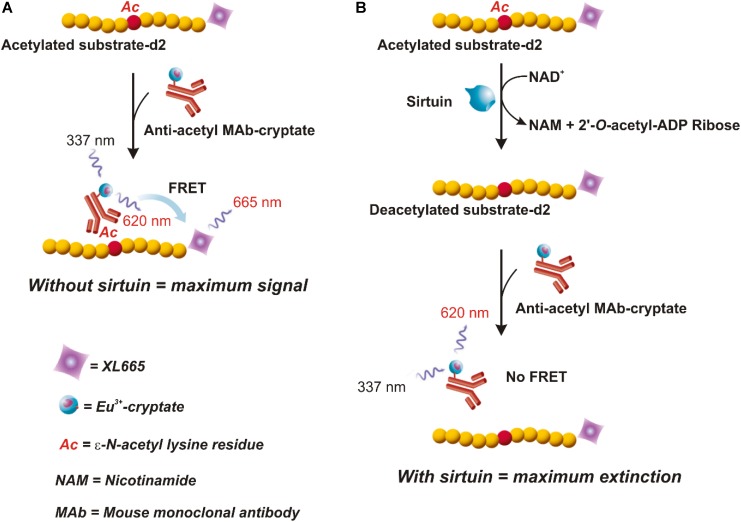
HTRF^®^ assay (HTRF^®^ SIRT1 Cisbio). In this assay an anti-acetyl specific Eu^3+^-Cryptate labeled antibody and an acetylated d2 substrate labeled with the phycobiliprotein pigment XL665 are used. In absence of sirtuin activity **(A)** the anti-acetyl Eu^3+^-Cryptate binds the acetylated substrate-d2. If the Eu^3+^-Cryptate is excited at 337 nm a FRET signal is obtained. On the contrary, in the presence of sirtuin **(B)** d2 labeled substrate is deacetylated thus preventing the binding with the antibody. In this case, no FRET is obtained. Therefore, the maximum signal is obtained in absence of sirtuin activity **(A)** and decreases proportionally to the deacetylation process **(B)**. Adapted from the Technical Manual of HTRF^®^ SIRT1 assay (Cisbio).

This assay combines standard FRET technology with time-resolved measurement of fluorescence. As shown in **Figure 2**, the assay uses a peptide containing a single acetylated lysine (substrate-d2) and bound to the XL665 fluorophore (λ_em_ = 665 nm), a phycobiliprotein pigment purified from red algae which acts as acceptor. The acetylated substrate-d2 can be recognized and bound by an anti-acetyl mouse monoclonal antibody labeled with a second fluorophore, the Eu^3+^-cryptate (λ_ex_ = 337 nm, λ_em_ = 620 nm), which is the donor. In the absence of sirtuin activity (A), the interaction between substrate and antibody brings the two fluorophores in close proximity; the excitation of Eu^3+^- cryptate triggers an energy transfer toward the XL665, which in turn emits specific fluorescence at 665 nm. Conversely, in the presence of sirtuins that deacetylate the substrate-d2, the interaction between substrate and antibody does not occur, resulting in loss of FRET and extinction in signal (B).

Measurements were performed by carrying out an enzymatic step followed by a detection step. During the enzymatic step, a mixture (volume of each well: 10 μL) consisting of the Enzymatic Buffer containing 1 mM DTT, 6 nM substrate-d2, 500 μM NAD^+^, was incubated at 25°C for 30 min in both the absence (No Enzyme Control, NoE) and the presence of sample, i.e., the sirtuin enzyme source (hSIRT1 or WM). For WM-sirtuin measurements, the reaction mixture also contained 0.1% Triton X-100, in order to lyse mitochondria and release mitochondrial sirtuin; it was preliminary checked that Triton X-100 had no significant effect on sirtuin activity detection system. Another substrate-d2-free mixture, consisting of the Enzymatic Buffer containing 1 mM DTT, 500 μM NAD^+^, 0.1% Triton X-100 (for WM-sirtuin measurements) was incubated for 30 min in the presence of sample (hSIRT1 or WM) and used as Negative Control (Neg). NoE and Neg controls were used to define the upper and lower limits of FRET signal, respectively.

In the detection step, the enzymatic reaction was stopped by the addition of 10 μl of anti-acetyl antibody dissolved in the Detection Buffer containing EDTA and NAM, a sirtuin inhibitor. The final volume of each well was 20 μL. After incubation from 5 h to overnight at 25°C, the fluorescence was measured at both 620 nm (emission of Eu^3+^-cryptate) and 665 nm (emission of XL665) by using a SpectraMax M5 Multimode Plate Reader (Molecular Devices, Wokingham, United Kingdom) and HTRF^®^ 96-well low volume plates (Cisbio). The 665 nm/620 nm fluorescence ratio (*Ratio*) was calculated and used to determine: (i) Δ*F*/Δ*F*_max_ according to the formula: (*Ratio_Sample_* - *Ratio_Neg_*)/(*Ratio_NoE_* - *Ratio_Neg_*); (ii) the substrate deacetylation (%) according to the formula: 100 - (*Ratio_Sample_*/*Ratio_NoE_* ⋅ 100).

For both hSIRT1 and WM sirtuin activity measurements, three different experiments were carried out. In each experiment determinations were carried out in triplicate by analysing different amounts of sample. A linear dependence of both Δ*F*/Δ*F*_max_ and substrate deacetylation (%) on the amount of sample was verified by linear regression analysis of data. Sirtuin activity was calculated from the substrate deacetylation (%) and expressed as mU (1 U = 1 pmol ⋅ min^-1^).

In experiments aimed at assessing possible modulation of sirtuin activity by resveratrol and quercetin, hSIRT1 and WM were incubated for 30 min with the compound at tested concentration before starting the enzymatic step. Resveratrol and quercetin were also added to NoE and Neg controls. Since resveratrol and quercetin were dissolved in DMSO, a constant volume of DMSO (0.25 μL) was maintained in the reaction mixture.

#### Marker Enzyme Assays

Phosphoenolpyruvate carboxylase (PEPC) and COX were assayed as marker enzymes of cytosol and mitochondria, respectively. PEPC activity was measured by monitoring spectrophotometrically the absorbance decrease at 340 nm due to NADH oxidation in the course of the PEPC/malate dehydrogenase coupled assay by means of a Perkin Elmer Lambda 45 UV/VIS spectrometer as reported in Soccio et al. (2013) and Trono et al. (2013). COX assay was checked oxygraphically by following oxygen consumption due to cytochrome c oxidation by means of a Gilson Oxygraph (model 5/6-servo Channel pH5) equipped with a Clark-type electrode (5331 YSI, Yellow Spring, OH, United States) as reported in Trono et al. (2013).

### Bioinformatic Analysis

Blastp search of the putative wheat sirtuins was performed using the Ensembl Plants^1^ website. Predictions of subcellular localization were made using iPSORT^2^, TargetP^3^, Predotar^4^ and MitoProt^5^ websites. Sequence alignments were obtained using the Vector NTI Suite software (version 11.5; Life Technologies).

## Results

In the present study, experiments were carried out to develop a new experimental approach able to reliably and accurately measure sirtuin activity in plant biological samples, by means of a protocol broadly applicable, technically simple and adaptable for high-throughput analysis. To achieve this goal, a combinatory application of enzymatic assays, differing in terms of substrates, principles of measurement, detection systems and quantification, was proposed. In particular, the luminescence SIRT-Glo^TM^ (Promega) and HTRF^®^-based SIRT1 (Cisbio) assays were applied. The SIRT-Glo^TM^ is based on detection of deacetylated peptides by three coupled sequential enzymatic events involving a substrate containing a ε-*N*-acetylated lysine residue bound to aminoluciferin. Briefly, upon acetyl group removal by sirtuin, aminoluciferin is released by specific proteolytic cleavage and then oxidized by firefly luciferase, thus inducing bioluminescence emission, whose magnitude is directly related to sirtuin deacetylase activity (for details see section “Materials and Methods” and **Figure 1**). A very different strategy characterizes the HTRF^®^ SIRT1 assay (Cisbio). Briefly, in this case, substrate deacetylation by sirtuin is detected by exploiting a FRET effect occurring between a fluorescent donor dye, consisting of Eu^3+^-cryptate bound to anti-acetyl mouse monoclonal antibody, and a fluorophore acceptor, represented by the XL665 phycobiliprotein connected to a substrate containing a single acetylated lysine (for details, see section “Materials and Methods” and **Figure 2**). In the absence of sirtuin (A), close proximity of two fluorophores due to substrate/antibody interaction ensures the maximum energy transfer from the excited Eu^3+^-cryptate toward XL665. Conversely, acetyl group removal by sirtuin activity prevents antibody binding to substrate, resulting in maximum extinction of FRET signal (B). Signal decrease is directly correlated to substrate deacetylation.

This methodological approach based on combinatory use of SIRT-Glo^TM^ and HTRF^®^ SIRT1 assays was first applied for studying native plant sirtuin activity in WM. Mitochondria were chosen since plant mitochondrial SRT2 isoforms have been demonstrated as key regulators of mitochondrial energy metabolism (König et al., 2014), and WM, in particular, are well-characterized from a bioenergetics point of view (Pastore et al., 1999, 2007; Laus et al., 2008, 2011; Soccio et al., 2010, 2013).

To this purpose, a preliminary investigation was carried out to identify sequences coding for putative sirtuins in bread wheat (*Triticum aestivum* L.), which is the species most phylogenetically related to durum wheat present in EnsemblPlants database. Based on a blastp search, using *Oryza sativa* SIR2b and *Arabidopsis thaliana* SRT2 proteins (Genbank accession numbers ABA95936 and AY045873, respectively) as query sequences, we identified: (i) a putative SRT1 (UniProt accession number A0A1D5UVD3) with a low homology (about 10%); (ii) two putative SRT2 (UniProt accession number A0A1D6S6B4 and A0A1D5YSJ0), encoded by two genes located on 5DL and 5AS chromosomes, respectively, and a putative SRT2 (UniProt accession number A0A1D6D8U6), whose gene position is unknown. All three putative SRT2 proteins show very high homology, up to about 87 and 55% with respect to OsSIR2b and AtSRT2, respectively (**Table 1**).

**Table 1:** Results of *Triticum aestivum* blastp search in EnsemblPlants database using OsSIR2b and AtSRT2 as query sequences.

UniProt accession number	OsSIR2b			AtSRT2		
		
	Score	*E*-value	% ID	Score	*E*-value	% ID
A0A1D6S6B4	1098	6.7E-141	86.6	794	5.0E-102	55.3
A0A1D5YSJ0	1066	3.1E-139	86.3	787	4.8E-101	55.1
A0A1D6D8U6	1066	3.1E-139	85.4	787	4.8E-101	54.8


**FIGURE 3:**
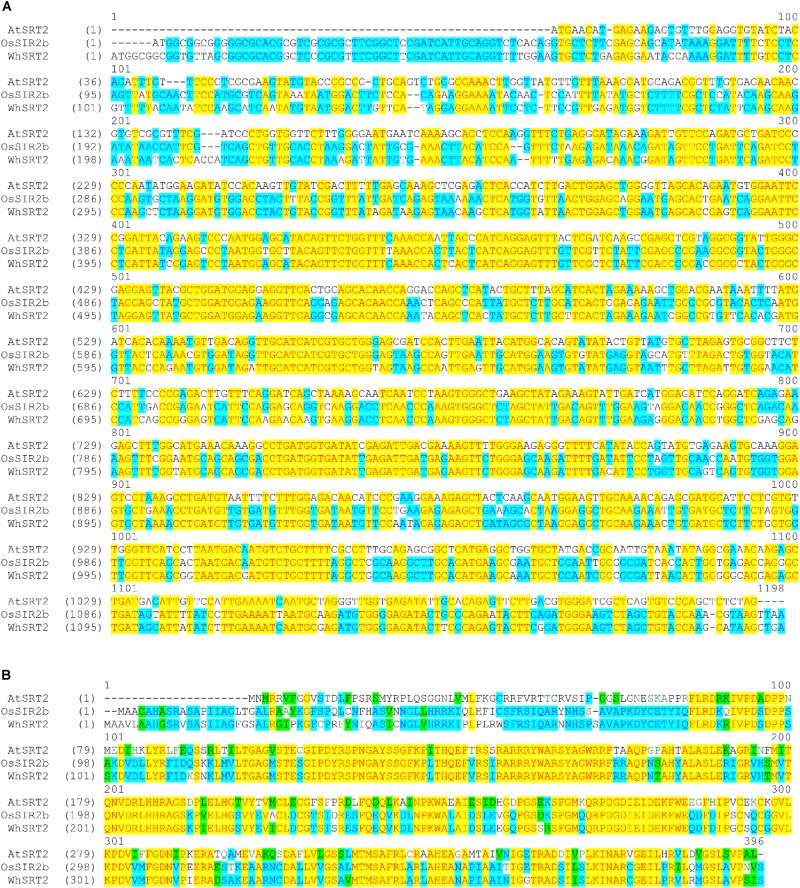
Alignment of the coding DNA sequences **(A)** and protein sequences **(B)** of AtSRT2, OsSIR2b and WhSRT2. Alignments were obtained using the Vector NTI Suite software (version 11.5; Life Technologies).

We focused on A0A1D5YSJ0 (which we named WhSRT2), since it is expected to be present in the tetraploid genome (AABB) of durum wheat. In **Figure 3** the alignment among AtSRT2, OsSIR2b and WhSRT2 at both nucleotidic (A) and aminoacid (B) sequences is reported. WhSRT2 is about 86% homolg to OsSIR2b at both nucleotidic and aminoacidic levels, and 61 and 55% homolg to AtSRT2 at nucleotidic and aminoacidic levels, respectively.

As for the comparison with respect to human mitochondrial sirtuins, WhSRT2 shares 35% amino acidic sequence identity with hSIRT4, showing mono-ADP-ribosyltransferase activity and undetectable deacetylase activity (Newman et al., 2012), and 19% identity with hSIRT3, the most active protein deacetylase in human mitochondria (Newman et al., 2012). These results are in agreement with that reported for AtSRT2 (König et al., 2014).

Bioinformatics analysis of subcellular localization of WhSRT2 in comparison with AtSRT2 and OsSIR2b by using the iPSORT, TargetP, Predotar, and MitoProt prediction software was also performed (**Table 2**). All the applied tools predicted a mitochondrial localization of AtSRT2; this result is in agreement with data from König et al. (2014) that demonstrated the exclusive AtSRT2 targeting to mitochondrial compartment. A prediction of mitochondrial/chloroplast localization was obtained for both OsSIR2b, as already reported by Chung et al. (2009), and WhSRT2. It should be outlined that for OsSIR2b an exclusive mitochondrial localization by using transient expression in tobacco BY2 cells has been demonstrated (Chung et al., 2009). Taken together, these results strongly suggest the existence of a putative mitochondrial SRT2 protein in wheat.

**Table 2:** Predicted subcellular localization of SRT2 from *Arabidopsis*, rice and wheat.

Name	Protein ID	Organism	Program			
			
			iPSORT^a^	TargetP^b^	Predotar^c^	MitoProt^d^
AtSRT2	AY045873^e^	*A. thaliana*	M	M	M	0.985
OsSIR2b^∗^	ABA95936^e^	*O. sativa*	M	C	P	0.917
WhSRT2	A0A1D5YSJ0^f^	*T. aestivum*	M	C	P	0.997


*^*a*^iPSORT (http://ipsort.hgc.jp/); ^*b*^TargetP (http://www.cbs.dtu.dk/services/TargetP/); ^*c*^Predotar (https://urgi.versailles.inra.fr/predotar/); ^*d*^MitoProt (https://ihg.gsf.de/ihg/mitoprot.html); ^*e*^GenBank accession numbers; ^*f*^UniProt accession number. M, mitochondria; C, chloroplasts; P, plastid. *^∗^Data from Chung et al. (2009).**

**FIGURE 4:**
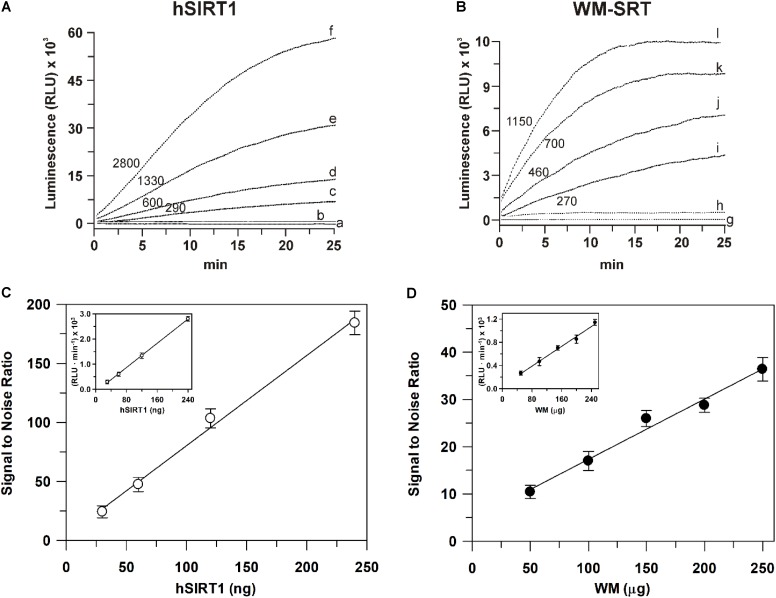
Luminescent signal mediated by the recombinant hSIRT1 **(A)** and WM-SRT **(B)** and dependence of deacetylase activity on hSIRT1 **(C)** and WM **(D)** amount. Sirtuin-mediated bioluminescent signal was monitored using the SIRT-Glo^TM^ assay (Promega) as described in Section “Materials and Methods” in presence of 30, 60, 120, and 240 ng of hSIRT1 (traces c, d, e and f, respectively) and 50, 100, 150 and 200 μg of WM (traces i, j, k, and l, respectively). In traces (b) and (h) measurements were carried out in presence of 50 mM NAM using 120 ng hSIRT1 and 150 μg WM, respectively. In traces (a) and (g) measurements were carried out after the boiling, at 100°C for 10 min, of 120 ng hSIRT1 and 150 μg WM, respectively. Numbers on the traces refer to the reaction rate expressed as RLU⋅min^-1^. In **(C,D)** sirtuin activity, expressed as “signal to noise ratio” and RLU⋅min^-1^ (insets), is reported as a function of hSIRT1 and WM amount, respectively. Data are reported as mean value ± SD (*n* = 3 different experiments).

As for sirtuin activity measurements, a first set of experiments was carried out to define experimental conditions suitable to assay this activity in WM. To this purpose, a comparison with a highly purified recombinant hSIRT1 was always carried out. As for measurements using the bioluminescent SIRT-Glo^TM^ assay, typical experimental traces are reported in **Figure 4A**. The addition of the SIRT-Glo^TM^ Reagent (containing acetylated substrate, NAD^+^, ATP, protease and luciferase, see section “Materials and Methods”) to the reaction mixture containing hSIRT1 caused an increase of bioluminescent signal until reaching a maximum steady-state value after about 25 min. In particular, an increasing steady-state signal was observed in the presence of increasing amounts (30, 60, 120, and 240 ng) of hSIRT1 (traces c, d, e, and f, respectively). Bioluminescent signal was completely abolished after boiling hSIRT1 at 100°C for 10 min (trace a), as well as in the presence of 50 mM NAM (trace b), the most potent physiological inhibitor of all sirtuin family enzymes (Bitterman et al., 2002; Jackson et al., 2003). These results demonstrate the correct functioning of the bioluminescent sirtuin/protease/luciferase coupled assay. As for WM, a similar behavior was observed. In particular, increasing amounts (50, 100, 150, and 200 μg) of WM proteins (**Figure 4B**, traces i, j, k, and l, respectively) caused an increase of bioluminescent steady-state signal. It should be underlined that in this case the reaction mixture also contained 0.1% Triton-X-100, 30 μM Ap5A, 4 μg oligomycin, in order to lyse mitochondria – so releasing mitochondrial sirtuin – and to avoid ATP consumption by mitochondrial metabolism. Similarly to hSIRT1, the activity was abolished in boiled WM and in presence of 50 mM NAM (traces h and g, respectively). Overall, these results demonstrate that the bioluminescence generation observed in WM is an enzyme-mediated reaction and it can be attributable to sirtuin activity. An increase of reaction rate (RLU⋅min^-1^) was also observed in the presence of increasing amounts of both hSIRT1 and WM proteins. A linear dependence of bioluminescent signal, expressed as signal to noise ratio (**Figure 4C**), was obtained on hSIRT1 amount in a wide protein range (25–250 ng). Similarly, the sirtuin activity in WM showed a linear response over protein amount ranging from 50 to 250 μg (**Figure 4D**). The result of **Figure 4C**, relative to hSIRT1, can also be used as a calibration curve to quantify the sirtuin activity in WM. Interestingly, WM displayed a significant sirtuin activity, equal to 166 ± 12 ng hSIRT1 eq. ⋅ mg^-1^ of WM protein. Linear responses over both hSIRT1 and WM proteins were also obtained when the bioluminescent signal was expressed as reaction rate (insets of **Figures 4C,D**). Interestingly, a statistically significant positive correlation was found between signal to noise values and reaction rates both in the case of hSIRT1 (*r* = 0.996, *P* < 0.001) and WM-SRT (*r* = 0.994, *P* < 0.001), respectively. These results show that reaction rate may be also used to quantify sirtuin activity, thus allowing a reduction of times of analysis.

By using the bioluminescent assay, another set of experiments was performed in order to assess whether sirtuin activity evaluated on WM is a true mitochondrial activity or a contamination due to other cellular fractions. At this purpose, the activities of sirtuin and of both PEPC and COX, marker enzymes of cytosol and mitochondria, respectively (Soccio et al., 2013; Trono et al., 2013), were evaluated in the total homogenate, in the combined pellets (containing nuclei) of the first and third centrifugations (see section “Materials and Methods”), and in the purified mitochondrial fraction. As shown in **Table 3**, PEPC specific activity strongly decreased in mitochondrial fraction; conversely, COX activity showed a strong specific activity enrichment of about 30-fold in mitochondrial fraction. These results confirm that the employed protocol is suitable to remove contamination due to cytosolic enzymatic activities and to obtain a highly purified mitochondrial fraction (Trono et al., 2013). As for sirtuin activity, no enrichment with respect to homogenate was observed in both pellet and mitochondrial fractions, thus suggesting that sirtuin activity is likewise present in both cellular compartments. Nevertheless, sirtuin enrichment in mitochondrial fraction was about twofold higher than that observed in pellet fraction. This probably depends on the higher cytosolic contamination in pellet fraction with respect to the mitochondrial one, as confirmed by the higher PEPC recovery. The activity detected in all tested fractions was found to be NAM-sensitive, thus confirming that the assayed activity is really due to a sirtuin activity. In particular, a complete inhibition by 100 mM NAM was observed in the pellet and WM fractions, whereas an about 90% inhibition was evaluated in the homogenate fraction. This demonstrates an exclusive sirtuin-dependent deacetylase activity in mitochondrial and pellet fractions, as well as only a very low (about 10%) deacetylase activity not attributable to sirtuins in the homogenate.

**Table 3:** Protein content and SRT, phosphoenolpyruvate carboxylase (PEPC) and cytochrome c oxidase (COX) activities in different fractions obtained in the course of WM purification.

Fraction	Protein (mg)	SRT			PEPC		COX	
		
		TA^a^	% Inhibition by NAM	(SA^b∗^, *E*^∗^)	TA^c^	(SA^d^, *E*)	TA^e^	(SA^f^, *E*)
Homogenate	1000	429000 ± 58000	90.2	(387, *1.00*)	52000 ± 4750	(52, *1.00*)	13800 ± 720	(13.8, *1.00*)
Pellet	35.1 ± 3.8	3300 ± 360	100	(94, *0.24*)	1544 ± 170	(44, *0.85*)	1035 ± 82	(29.5, *2.14*)
Mitochondria	4.1 ± 0.3	720 ± 85	100	(176, *0.45*)	49 ± 6	(12, *0.23*)	1566 ± 185	(382, *28*)



**^*a*^ng of hSIRT1 eq.; ^*b*^ng hSIRT1 eq. ⋅ mg^-*1*^ protein; ^*c*^nmol of NADH oxidized ⋅ min^-*1*^; ^*d*^nmol NADH oxidized ⋅ min^-*1*^. mg^-*1*^ protein; ^*e*^nmol of oxygen taken up ⋅ min^-*1*^; ^*f*^ nmol of oxygen taken up ⋅ min^-*1*^ ⋅ mg^-*1*^ protein; ^∗^values are obtained by using the NAM-sensitive activity. Protein content and enzymatic activities were carried out as reported in Section “Materials and Methods”. For each enzyme, total activity (TA) and specific activity (SA) with respect to 1 g homogenate proteins, as well as the enrichment (E) of SA with respect to homogenate, are reported for all tested fractions. For SRT activity, % of inhibition by 100 mM is also reported. Data are expressed as mean value ± S.D. (n = 3 different experiments).**

Taken together, these results support even more the choice of WM as a good plant system to study native plant sirtuin activity.

**FIGURE 5:**
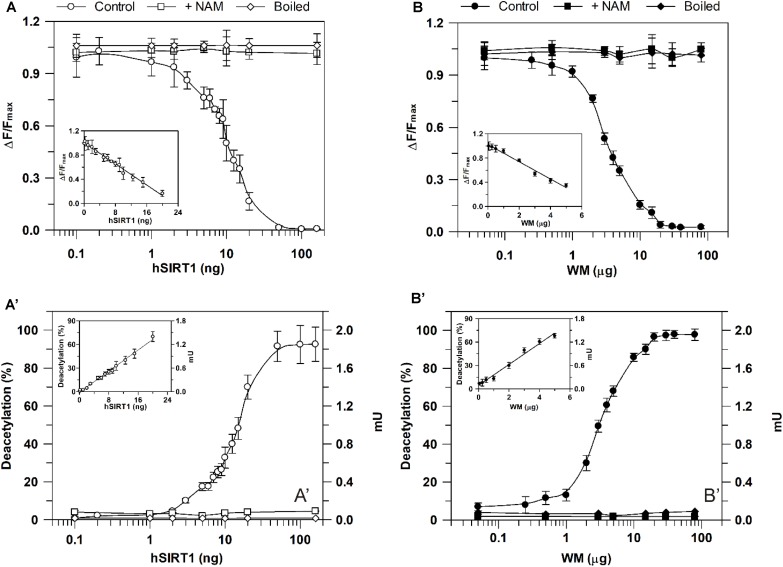
Dependence of deacetylase activity, evaluated using he HTRF^®^-based method, on hSIRT1 **(A,A′)** and WM **(B,B′)** amount. Sirtuin activity was monitored using the HTRF^®^ SIRT1 assay (Cisbio) as described in Section “Materials and Methods” using different hSIRT1 **(A,A′)** and WM **(B,B′)** amounts, in absence (control) and in presence of 50 mM NAM, as well as after sample boiling (100°C for 10 min), respectively. Data are expressed as Δ*F*/*F*_max_ **(A,B)** and as both deacetylation (%) and mU **(A′,B′)**. In the insets, protein ranges, in which a linear dependence was obtained, are shown. All data are reported as mean value ± SD (*n* = 3 different experiments).

As for sirtuin activity measurements using the HTRF^®^ assay, experiments were carried out by running enzymatic reaction for 30 min in the presence of increasing hSIRT1 amounts (0.1–160 ng). As shown in **Figure 5A**, Δ*F*/*F*_max_ parameter was found to decrease with increasing amounts of hSIRT1 (control). As above reported, this depends on the hSIRT1-mediated deacetylation of the substrate-d2, causing a loss of FRET signal with a consequent reduction of Δ*F*/*F*_max_ parameter (see also section “Materials and Methods” and **Figure 2**). Δ*F*/*F*_max_ parameter was found to remain constant at the maximum value after boiling hSIRT1 at 100°C for 10 min, as well as in the presence of 50 mM NAM. The dependence of hSIRT1 activity on protein amount was also reported both in terms of percentage of deacetylated substrate and mU (**Figure 5A**′). As expected, an opposite behavior was obtained with respect to Δ*F*/*F*_max_ parameter. In particular, a highly statistically significant inverse correlation (*r* = -0.987, *P* < 0.001) was found between Δ*F*/*F*_max_ parameter and (%) deacetylation (or mU values), thus indicating the correct functioning of the assay. A linear dependence of Δ*F*/*F*_max_ parameter (inset of **Figure 5A**), as well as of percentage of deacetylated substrate and mU (inset of **Figure 5A**′), on hSIRT1 protein amount was obtained in 0.1–20 ng range. It should be underlined that, in parallel, a time course study was performed in which the enzymatic step was carried out for increasing incubation times ranging from 15 to 240 min; a linear dependence from 15 to 90 min was found, thus confirming that 30 min is an appropriate incubation time for the enzymatic step (data not shown).

A profile similar to hSIRT1 was obtained for Δ*F*/*F*_max_ parameter in the presence of WM proteins ranging from 0.05 to 80 μg WM (**Figure 5B**, control). It should be considered that, in order to allow the complete lysis of the organelles and make free the sirtuin activity, in this case the reaction mixture contained 0.1% Triton-X-100. Similarly to hSIRT1, activity was abolished in boiled WM and in the presence of 50 mM NAM. An opposite profile with respect to Δ*F*/*F*_max_ parameter was obtained for WM-sirtuin expressed as percentage of deacetylated substrate and mU (**Figure 5B**′). Also in the case of WM, a significant inverse correlation (*r* = -0.999, *P* < 0.001) was found between Δ*F*/*F*_max_ parameter and (%) deacetylation (or mU values), thus confirming the correct functioning of the assay also in purified mitochondrial fraction. Similarly to hSIRT1, WM-SRT activity showed a linear response over protein amount ranging from 0.05 to 5 μg, evaluated as Δ*F*/*F*_max_ (inset of **Figure 5B**), as well as percentage of deacetylated substrate and mU (inset of **Figure 5B**′). As already reported for the bioluminescent assay, these results demonstrate that FRET signal changes observed in WM can be attributable to sirtuin activity. Interestingly, WM-SRT activity equal to 268 ± 10 mU ⋅ mg^-1^ of WM protein was measured.

**FIGURE 6:**
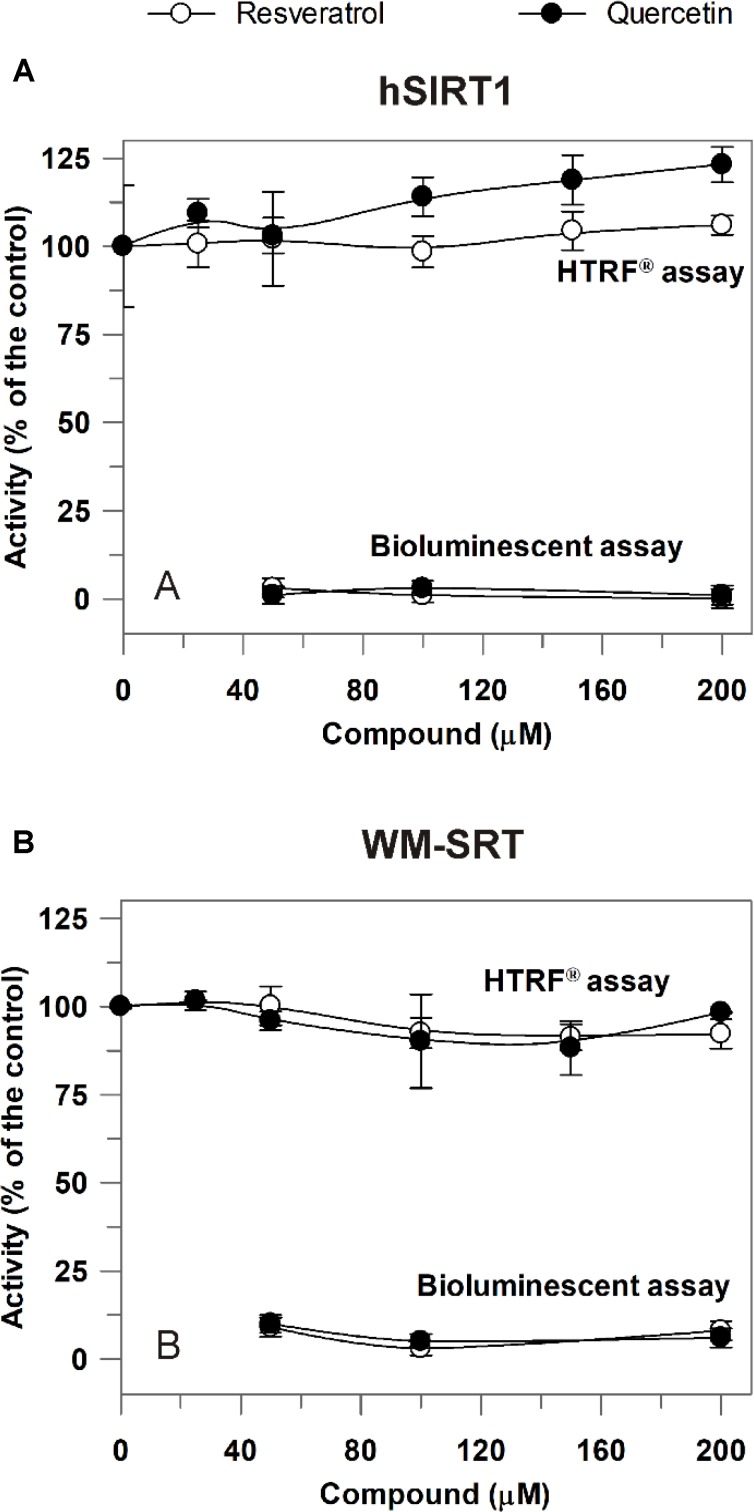
Effect of resveratrol and quercetin on hSIRT1 **(A)** and WM-SRT **(B)** activities evaluated by both the luminescent and HTRF^®^ assays. **(A)** hSIRT1 activity was evaluated by HTRF^®^ and luminescent assays, as reported in Section “Materials and Methods,” by using 15 and 120 ng hSIRT1, respectively. **(B)** WM-SRT activity was evaluated by HTRF^®^ and bioluminescent assays, as reported in “Materials and Methods”, by using 3 and 150 μg WM, respectively. Measurements were carried out in the absence (control) and in presence of different concentrations of resveratrol and quercetin. Data are expressed as % of the control and reported as mean value ± SD (*n* = 3 different experiments).

The newly developed approach was then applied to study modulation of sirtuin activity by resveratrol and quercetin, known as sirtuin activating compounds (Howitz et al., 2003), but whose activation mechanism has raised controversies (Hubbard and Sinclair, 2014). Also in this case, a comparison between hSIRT1 and WM-SRT activities was made. As shown in **Figure 6A**, in 50–200 μM range a complete inhibition on hSIRT1 by both resveratrol and quercetin was observed when the bioluminescent assay was applied. A complete inhibition was observed also by using the non-acetylated control substrate (data not shown), thus allowing to exclude a direct inhibition of resveratrol and quercetin on hSIRT1; conversely, the observed effect can depend on the capability of resveratrol and flavonoids to inhibit the firefly luciferase activity (Bakhtiarova et al., 2006; Auld et al., 2008; Zhang et al., 2017). A similar behavior was observed also for WM-SRT (**Figure 6B**). Interestingly, a different response was observed when the HTRF^®^ assay was used. In this case, the effect of both phenolic compounds was evaluated in 25–200 μM range. As shown in **Figure 6A**, no effect was observed on hSIRT1 in the presence of resveratrol and only a slight increase, up to about 20%, was measured in the presence of 200 μM quercetin. As for WM (**Figure 6B**), no effect on sirtuin activity was observed both in presence of resveratrol and quercetin.

## Discussion

### Novel Approach for Sirtuin Activity Measurements: Use of Methods Based on Different Rationales and Comparison With hSIRT1

This study arises in an attempt to overcome the difficulty of carrying out direct sirtuin activity assessment in plant cellular systems. To this purpose, a novel approach for measuring sirtuin activity in plant cell extracts that combines the use of multiple appropriately selected methods was proposed. The main requirement for choosing the assays to be combined is that methods are based on different reaction mechanisms and employ different substrates, experimental conditions, detection methodologies and quantification modes. Moreover, the following criteria should also be met: (i) assays should use simple protocols involving a readily available instrumentation, easily applicable to cell extracts and able to provide good performance in terms of accuracy and reproducibility; (ii) they should be as far as possible able to avoid potential artifacts due to interfering molecules in the reaction mixture/biological sample.

With respect to the last point, intense debate on the effects of some putative STACs has arisen from the use of fluorimetric assays employing acetylated substrate peptides conjugated to AMC or carboxytetramethylrhodamine (TAMRA) fluorophores. By using the commercially available Fluor de Lys kit based on AMC-fluorescent assay, some physiological STACs were identified, among which the most effective was resveratrol (Howitz et al., 2003). Subsequently, by using TAMRA-tagged substrates several synthetic STACs were also discovered (Milne et al., 2007; Dai et al., 2010; Hubbard et al., 2013). On the other hand, by using isotopic methods in comparison with Fluor de Lys assay, two independent research groups demonstrated that resveratrol is not a direct activator of SIRT1, being the activation completely dependent on the presence of covalently attached fluorescent moieties in the peptide substrate (Borra et al., 2005; Kaeberlein et al., 2005). The fluorophore-specific activation of SIRT1 by resveratrol was also demonstrated by means of a fluorimetric assay based on quantification of remaining NAD^+^ after deacetylation (Feng et al., 2009), as well as by direct HPLC detection and quantification of acetylated/deacetylated TAMRA-p53-derived peptide substrates (Pacholec et al., 2010). On the contrary, another report showed that some STACs activate the deacetylation of unlabeled peptides composed only of natural amino acids (Dai et al., 2010). A subsequent study demonstrated that *in vitro* SIRT1 activation by resveratrol and synthetic STACs requires hydrophobic residues in specific positions with respect to the acetylated lysine in some native peptide substrates (Hubbard et al., 2013). Finally, using peptide microarrays a separate research group demonstrated that effects of resveratrol are substrate sequence-selective, with activation occurring only with a few physiological acetylation sites, but not with other native sequences (Lakshminarasimhan et al., 2013).

In the light of these issues, an adequate combination of enzymatic methods deeply differing for chemical basis and rationale may represent an effective strategy for revealing any interfering compounds and unmasking possible false positive/negative hits, so allowing a correct data interpretation.

In particular, in the present study sirtuin activity was evaluated by combinatory use of the luminescence SIRT-Glo^TM^ (Promega) and HTRF^®^-based SIRT1 (Cisbio) assays. Both methods have the advantage of being a highly sensitive assay of sirtuin activity. Nevertheless, for the SIRT-Glo^TM^ assay the activity assessment is obtained by measuring bioluminescence emission associated to aminoluciferin oxidation occurring in three coupled enzymatic events involving sirtuin/protease/firefly luciferase; moreover, sirtuin activity may be quantified in terms of arbitrary steady state signal. The advanced HTRF^®^ SIRT1 assay directly measures the FRET signal level depending on the proximity between Eu^3+^-cryptate donor and XL665 acceptor fluorophores (bound to anti-acetyl antibody and acetylated substrate, respectively), that in turn depends on substrate deacetylation by sirtuin activity. With respect to bioluminescence assay, the HTRF^®^ assay shows the advantage (i) of using the ratio between the donor and acceptor emission signals able to provide useful information for the recognition of interfering substances, and (ii) of allowing a quantification of sirtuin activity in terms of enzymatic units. It should be outlined that both used assays can easily be performed by using microplate readers and applied in high-throughput analysis.

In addition to the appropriate selection of assays, the newly proposed approach for sirtuin activity determination in biological samples involves the parallel measurement of a highly purified recombinant sirtuin isoform. Firstly, the use of a recombinant isoform allows verifying the correct functioning of detection systems. Moreover, the similar behavior observed in both purified sirtuin and biological sample under the same experimental conditions strongly suggests the existence of sirtuin activity also in the tested sample. In the case of bioluminescence assay and for all the assays providing an arbitrary quantification of sirtuin activity, the parallel measurement of a recombinant enzyme may be used as an internal calibration, able to provide a relative quantification of sirtuin activity of the tested sample. This may allow cross comparison among results obtained from different biological systems, experimental conditions, research groups and laboratories. In particular, as reported in Introduction, hSIRT1 was chosen since it is the best-studied sirtuin isoform.

### WM as a Plant System for Evaluating Sirtuin Activity

The combinatory application of bioluminescence SIRT-Glo^TM^ and HTRF^®^ SIRT1 assays was used for the first time for studying sirtuin activity in WM, in comparison with the purified recombinant hSIRT1. The interest in mitochondrial sirtuins arises from their relevant role in fine-tuning of mitochondrial energy metabolism recently reported for *At*SRT2 protein isoforms, able to catalyze the specific deacetylation of ATP/ADP carrier and ATP synthase (König et al., 2014).

The choice of WM takes into account different aspects. Firstly, the existence of a putative mitochondrial sirtuin activity is expected in wheat. This is in the light of recent literature data demonstrating the existence of plant mitochondrial sirtuin in the dicotyledonous *Arabidopsis thaliana* (König et al., 2014), as well as in a monocotyledonous cereal species phylogenetically far from *Arabidopsis*, but closely related to wheat, such as rice (Chung et al., 2009). In fact, by means of an *in silico* analysis we identified in wheat a SRT2 protein having (i) highly homology to SRT2 from rice and *Arabidopsis* and (ii) a very high probability to localize into the mitochondria, as supported by bioinformatics tools. These results strongly support the occurrence of sirtuin activity in WM.

Another aspect that should be considered is that highly pure, intact and functional mitochondria may be isolated with high yield from wheat seedlings (Pastore et al., 1999). Moreover, by measuring sirtuin activity, in comparison with cytosol and mitochondria marker enzymes, in different fractions obtained in the course of WM isolation, the highly purified mitochondrial fraction resulted (i) characterized by completely NAM-sensitive deacetylase activity with an enrichment about twofold higher than that observed in nuclear fraction (one of the major sites of sirtuin activity), and (ii) affected only by a negligible cytosolic contamination compared to nuclei. In particular, a significant sirtuin activity equal to 268 ± 10 mU ⋅ mg^-1^ protein and 166 ± 12 ng hSIRT1 eq. ⋅ mg^-1^ protein was measured in WM, as evaluated by using HTRF^®^ and the bioluminescence assays, respectively.

High recovery of mitochondrial proteins guaranteed by WM isolation protocol and significant sirtuin activity levels in purified mitochondrial fraction allow performing a large number of measurements from the same preparation (about 15–50 in RLU and 500-2000 in HTRF^®^). Moreover, for both the bioluminescence and HTRF^®^ sirtuin assays the use of microplate readers allows for screening applications, thus strongly increasing the number of processed samples and reducing analysis times and assay cost per sample, also maintaining high repeatability of the results.

Taken together, these results strengthen the use of WM as a well-characterized good plant system for studying plant mitochondrial sirtuin.

An interesting aspect should be emphasized. By using an appropriately developed experimental strategy, the present study reports the first measurement of catalytic activity of a “native” plant sirtuin, i.e., the first direct assessment of intracellular sirtuin activity and, in particular, within a subcellular system represented by purified mitochondrial fraction. In this regard, König et al. (2014) reported assessment of plant mitochondrial sirtuin activity, but, in this case, deacetylase activity of *Arabidopsis* SRT2 proteins was measured using recombinant proteins and indirectly demonstrated by the increased lysine acetylation levels of specific mitochondrial target proteins in *srt2* loss-of-function mutants.

Interestingly, we found a high deacetylase activity in WM, although WhSRT2 is much more homologous to mitochondrial hSIRT4 (showing undetectable deacetylase activity) than hSIRT3 (being the most active mitochondrial deacetylase). This result is in agreement with that reported for AtSRT2 (König et al., 2014).

In light of its highly specific activity, an important physiological role can be suggested for WM-sirtuin, in analogy with key role of *Arabidopsis* SRT2 in fine regulation of energy metabolism (König et al., 2014). The possible implications of WM-sirtuin activity in post-translational regulation of some transport pathways, extensively studied in WM and having a role in controlling ROS generation and mitochondrial bioenergetics under environmental/oxidative stress conditions (Trono et al., 2014, 2015 and refs therein), merit future investigations.

### Study of Modulation of WM-Sirtuin Activity by Resveratrol and Quercetin

The developed approach was applied for studying the effects of two phenols – such as resveratrol and quercetin – on both hSIRT1 and WM-SRT activities. These compounds have been reported as potent hSIRT1 activators (Howitz et al., 2003), but with respect to their activation mechanism inconsistent and conflicting findings have been reported (Hubbard and Sinclair, 2014). The combinatory use of bioluminescence/HTRF^®^ sirtuin assays proved to be crucial in identifying the false inhibition effect of both tested compounds shown by SIRT-Glo^TM^ assay, which actually may be attributed to the capability of resveratrol and flavonoids to inhibit the firefly luciferase activity (Bakhtiarova et al., 2006; Auld et al., 2008; Zhang et al., 2017). Interestingly, by using HTRF^®^ assay no effect of resveratrol was observed on both WM-SRT and hSIRT1 activities, while only a slight increase, up to about 20%, of hSIRT1 activity by quercetin was observed, much lower than that reported in previous studies using Fluor de Lys assay (Howitz et al., 2003; de Boer et al., 2006). So, although a fluorophore-labeled substrate was used in HTRF^®^ assay, we did not found any activation of hSIRT1 by resveratrol, unlike what was observed in previous studies reporting activation using AMC- or TAMRA-tagged substrates but not unlabeled peptides (Borra et al., 2005; Kaeberlein et al., 2005; Pacholec et al., 2010). The lack of a significant effect of both resveratrol and quercetin on hSIRT1 observed in our study may be explained in the light of findings of Lakshminarasimhan et al. (2013), showing either SIRT1 activation or no effect or inhibition by resveratrol depending on native peptide sequences used as substrate to measure activity.

Interestingly, under our experimental conditions a lack of substantial effect of resveratrol and quercetin was observed on both hSIRT1 and WM-SRT. Considering the high phylogenetic distance between wheat and human species, this result may allow generalizing effects of these compounds to sirtuins from different plant sources under the same experimental conditions. This allows hypothesizing the use of WM to obtain preliminary information about modulation of other plant sirtuins characterized by very low and not easily detectable activity. Overall, these results strongly support the use of WM as a good system to study plant sirtuin activity.

## Conclusion

Results of this study demonstrate that, by combining the use of two different enzymatic assays to the comparative measurement of activity of a highly purified recombinant enzyme (hSIRT1), a reliable and reproducible quantification of native sirtuin activity in plant biological samples can be achieved. For assessing sirtuin activity in plant extracts, a good system has been identified in WM, able to be isolated with high purity and good yields and showing high sirtuin specific activity. The proposed methodology using WM and microplate readers can be qualified for application to large-scale high-throughput screening. In the light of this, the newly developed approach may represent an excellent tool to assess plant sirtuin activity changes in cellular/subcellular systems under different experimental conditions, thus allowing to gain more biologically relevant insights into its physiological role and modulation compared to that obtained using pure/recombinant enzymes.

Moreover, when combined with gene-expression and knockout lines-based studies, the proposed approach may contribute to improve understanding of plant sirtuin function.

## Author Contributions

MS, ML, and MA performed the experiments and processed the experimental data. MS and ML wrote the manuscript with support from MA. DP conceived the study, supervised the research, and co-wrote the manuscript.

## Conflict of Interest Statement

The authors declare that the research was conducted in the absence of any commercial or financial relationships that could be construed as a potential conflict of interest.

## Footnotes

1 http://plants.ensembl.org/index.html

2 http://ipsort.hgc.jp/

3 http://www.cbs.dtu.dk/services/TargetP/

4 https://urgi.versailles.inra.fr/predotar/

5 https://ihg.gsf.de/ihg/mitoprot.html

## Abbreviations

AMC: 7-amino-4-methylcoumarin
Ap5A: P^1^,P^5^-di(adenosine-5′) pentaphosphate
BSA: bovine serum albumin
COX: cytochrome c oxidase
DMSO: dimethyl sulfoxide
FRET: Fluorescence Resonance Energy Transfer
hSIRT1: human SIRT1 isoform
HTRF^®^: Homogeneous Time Resolved Fluorescence
NAM: nicotinamide
PEPC: phosphoenolpyruvate carboxylase
PVP: polyvinylpyrrolidone
RLU: Relative Luminescence unit
SIRT: mammalian sirtuin isoforms
SRT: plant sirtuin isoforms
STAC: SIRT1-activating compound
TAMRA: carboxytetramethylrhodamine
WM: wheat mitochondria
XL665: phycobiliprotein pigment purified from red algae
